# Privacy Protection and Intrusion Detection System of Wireless Sensor Network Based on Artificial Neural Network

**DOI:** 10.1155/2022/1795454

**Published:** 2022-06-22

**Authors:** Lusheng Shi, Kai Li

**Affiliations:** School of Information Engineering, Suqian University, Suqian 223800, China

## Abstract

With the increasing openness and development of network technology, the network based on the wireless sensor network system has increasingly become an important tool for human social life and production, but it also brings some network security problems. Among them, focus is on network privacy disclosure and foreign intrusion and the research of intrusion detection and privacy protection has increasingly become an important topic of network security. This paper deeply studies the wireless sensor network system based on neural network on the basis of traditional privacy protection and intrusion detection system. Firstly, it applies particle swarm optimization algorithm and constructs a wireless sensor network intrusion detection system based on particle swarm optimization algorithm. The system includes important modules such as data extraction, data analysis, data feedback, and auxiliary decision-making. Compared with other algorithms, particle swarm optimization algorithm does not rely on problem information. It mainly uses real numbers to solve, so the algorithm has strong universality. At the same time, its corresponding principle is simple and easy to implement and less parameters need to be adjusted. Compared with other algorithms, particle swarm optimization algorithm has fast convergence speed and little memory requirement for the computer. At the same time, this paper uses the leap of particle swarm optimization algorithm to make it easier to find the global optimal solution. At the corresponding level of wireless sensor network privacy protection, based on the original data aggregation privacy protection scheme, this paper proposes a privacy protection scheme based on polynomial regression and a user privacy protection scheme based on the same state encryption, which further improves the security of privacy protection and facilitates the management of information. To realize the integrity of user privacy information protection, this paper realizes the decryption of data based on the correlation between binary metadata and compares the corresponding decrypted data with aggregated data, so as to complete the integrity of privacy data protection. The experimental results show that the binary metadata correlation decryption method proposed in this paper and the introduction of the corresponding particle swarm optimization algorithm improve the stability of the system by about 10%, the corresponding system security by a positive proportion, and the integrity of private data by about 10%; therefore, the algorithm proposed in this paper has obvious advantages.

## 1. Introduction

On the one hand, users' awareness of network security is weak, the corresponding system is not perfect, and the corresponding management and detection system are not perfect, which makes a large number of network viruses and security problems rush into the Internet system, endangering the privacy and security of Internet users [1–3]; on the other hand, network security, privacy protection, and intrusion detection technology are relatively lagging behind, and there are still serious technical problems in the development of corresponding security protection and system security. With network and the rapid development of network sharing and other related services, the object and scope of network technology services are further expanded, and the resulting network security risks are also further expanded [4, 5]. Comprehensive analysis shows that the main hidden dangers of current network security include the design defects of the physical level of network structure, the contradictions existing in the openness characteristics of the network, and the decreasing difficulty of traditional network password decoding. At present, the main intrusion methods of network wireless sensor networks mainly include distributed network intrusion means, target machine attack means, denial of service attack means, and corresponding system attack means [6, 7]. In addition to the problems of intrusion detection technology, the current privacy protection technology of wireless sensor networks is also threatened by the energy, calculation, and storage of wireless sensor network nodes. The unreliability of its channels and the variability of multi-hop transmission also make the corresponding wireless sensor networks prone to channel information theft and information leakage during information transmission [8]. Therefore, if the information network system of wireless sensor network wants to develop rapidly, it must solve the corresponding problems of privacy protection and intrusion detection.

Traditional wireless sensor network intrusion detection methods mainly collect, analyze, and detect any attack that attempts to damage the computer system or wireless sensor network system from the computer system or the corresponding wireless sensor network system, so as to ensure the integrity, confidentiality, and corresponding availability of computer resources. As a remedy for the technical deficiency of traditional firewall, intrusion detection technology can supervise external users in real time [9, 10]. The current wireless sensor network intrusion detection technology mainly has the following problems: the limited scalability of intrusion detection system, which is often limited to a specific environment and does not have universality [11, 12]; the corresponding intrusion detection system has a serious problem of resource dissipation; the corresponding traditional intrusion detection system has poor performance, and the corresponding data processing performance is not high, which cannot meet the real-time requirements of intrusion detection system. In the corresponding wireless sensor network privacy protection scheme, it mainly includes user data-oriented privacy protection and context data-oriented privacy protection. The corresponding user data-oriented privacy protection mainly includes data aggregation-oriented privacy protection and corresponding user data query privacy protection, and the corresponding context-oriented data privacy protection mainly includes the corresponding data location-oriented privacy protection and data time-oriented privacy protection [13, 14]. The privacy protection means of the corresponding wireless sensor network system mainly include encrypting the corresponding user data, hiding the corresponding user data, source node location information protection technology, source node time information protection technology, protection technology based on disturbed data, multi-party technology based on data security, polynomial regression technology based on data, and confidentiality technology based on the same data state [15]. The existing intrusion detection systems are similar, and the analysis and research of most intrusion detection systems will have the following disadvantages: the detection speed of the existing intrusion detection system is much lower than the network transmission speed, resulting in false positives; the combination of intrusion detection products and other network security products, that is, information exchange during the period, cooperates to find and block attacks; the network-based intrusion detection system cannot detect the encrypted data flow and the data flow under the switching network, and its own construction is vulnerable to attack, intrusion detection system architecture.

This paper analyzes and studies the privacy protection and intrusion technology of wireless sensor network system. Based on the existing related problems, this paper deeply studies the wireless sensor network system based on neural network on the basis of traditional privacy protection and intrusion detection system. Firstly, it applies correlation algorithm to construct a wireless sensor network. The system includes data extraction, data analysis, and important modules such as data feedback and auxiliary decision-making; at the privacy protection, based on the original data aggregation privacy protection scheme, this paper proposes a privacy protection scheme based on polynomial regression and a user privacy protection scheme based on the same state encryption, which further improves the security of privacy protection and facilitates the management of information. In order to realize the integrity of user privacy information protection, it realizes the decryption of data above on the correlation between binary metadata and compares the corresponding decrypted data with aggregated data, so as to complete the integrity of privacy data protection. The results show that the privacy protection and intrusion detection system designed in this paper have high practical value.

The main research contents of this paper are as follows: the third section of this paper will focus on the analysis of the wireless sensor network privacy protection and intrusion detection system scheme proposed in this paper. At the same time, it will specifically analyze the intrusion detection technology of wireless sensor network based on particle swarm optimization algorithm, as well as the privacy protection scheme based on polynomial regression and homomorphic encryption; in the fourth section, the sample data will be verified and the experimental data will be analyzed; finally, this paper is summarized.

## 2. Related Research: Research Status of Privacy Protection and Intrusion Detection System of the Wireless Sensor Network System Based on Artificial Neural Network

A large number of research institutions and researchers have analyzed and verified the privacy protection scheme and the current wireless sensor network system, and given the corresponding solutions. At the combination level of artificial neural network and intrusion detection technology, relevant European researchers tend to use artificial intelligence technology combined with encryption technology to monitor and analyze intrusion objects. The main technology includes random key management algorithm, which mainly adopts the random allocation of secret materials [16, 17]; corresponding researchers proposed hybrid encryption algorithm based on the above random key allocation algorithm, which is mainly based on elliptic curve cryptography algorithm and advanced encryption standard algorithm for hybrid processing, so as to improve the performance of intrusion detection system [18, 19]; relevant researchers combined with artificial intelligence algorithm proposed the intrusion detection technology of trust-based adaptive confirmation, which uses the trust algorithm of Kalman filter [20]; relevant American researchers propose refining the data model by constantly searching the corresponding relationship between data and reduce the corresponding redundant knowledge, so as to further reduce the information overload and improve the decision-making ability of the corresponding information model [21, 22]. In the corresponding privacy protection strategy of wireless sensor networks, its main algorithms include data privacy protection scheme based on data disturbance, data privacy protection scheme based on secure multi-party technology, privacy data protection scheme based on polynomial regression, and encryption technology based on the same data state [23, 24]. On this basis, relevant technicians in the USA have proposed the idea of cluster-based and the related algebraic properties of polynomials for corresponding data aggregation and corresponding privacy protection. On the basis of this technology, some researchers have carried out a large number of optimization processing based on calculation and corresponding overhead level [25–28]; relevant Japanese researchers have proposed a tree-based multivariate polynomial data regression algorithm, which sends polynomial technology to the base station for aggregation operation, so as to reduce the corresponding data traffic and protect the privacy of data [1, 29].

## 3. Privacy Protection and Intrusion Detection System Design of the Wireless Sensor Network System Based on Particle Swarm Optimization Algorithm

**Figure 1:**
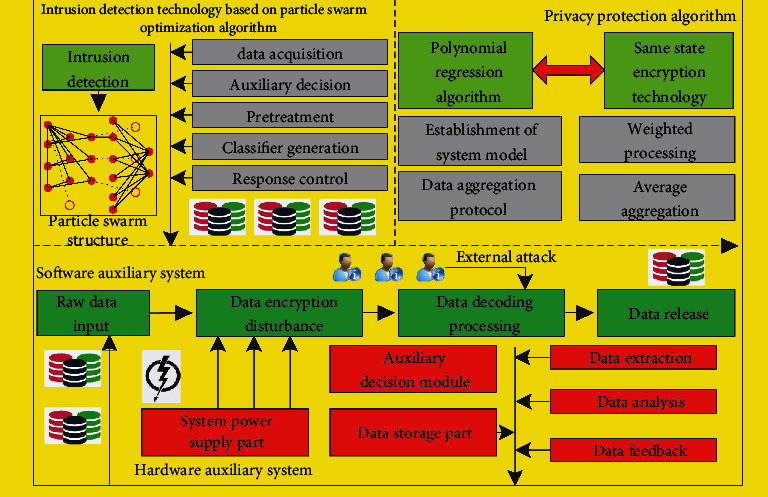
Principle frame diagram of the privacy protection and intrusion detection system of wireless sensor network system based on particle swarm optimization.

This section mainly analyzes and studies the key algorithms of privacy protection and intrusion detection system based on wireless sensor network system under particle swarm optimization. The corresponding system design framework is shown in Figure 1. From Figure 1, the corresponding system mainly includes two parts of software algorithm architecture, namely, the software algorithm architecture of intrusion detection technology based on particle swarm optimization algorithm and the privacy protection algorithm architecture based on polynomial regression and same state encryption technology. The corresponding software algorithm architecture of intrusion detection technology based on particle swarm optimization algorithm mainly includes data acquisition module, auxiliary decision-making module, corresponding preprocessing module, classifier generation module, and response control module. The corresponding data acquisition module mainly obtains the original data, preliminarily refines and processes the corresponding original data, and sends such data to the preprocessing module for processing, so as to capture the network data packets transmitted by channel, and then analyze the corresponding data packets for subsequent processing and analysis; the corresponding auxiliary decision-making module mainly takes the corresponding network data as the corresponding data source, so as to analyze the amount of network data and make corresponding decisions; based on the corresponding particle swarm optimization algorithm, search the global corresponding information and make decision-making judgment and analysis, so as to realize the detection and processing of intrusion and other related objects. The corresponding privacy protection algorithm architecture based on polynomial regression and same state encryption technology mainly includes polynomial regression algorithm and same state encryption algorithm. The corresponding polynomial regression algorithm includes the establishment of system model, the formulation of polynomial data aggregation protocol, and the corresponding performance evaluation, and the corresponding same state encryption algorithm mainly includes the weighted average aggregation scheme algorithm and the setting and processing of corresponding system parameters. There are few corresponding contents in the corresponding hardware part. The main modules include power supply and power management part, data storage part, etc. At the corresponding algorithm operation level, the main principles and ideas are as follows: particle swarm optimization algorithm is applied to intrusion detection system, and a wireless sensor network intrusion detection system based on particle swarm optimization algorithm is constructed. The system includes important modules such as data extraction, data analysis, data feedback, and auxiliary decision-making; at the corresponding level of its privacy protection, based on the original data aggregation privacy protection scheme, this paper proposes a privacy protection scheme based on polynomial regression and a user privacy protection scheme based on the same state encryption, which further improves the security of privacy protection and facilitates the management of information. To realize the integrity of user privacy information protection, this paper realizes the decryption of data based on the correlation between binary metadata and compares the corresponding decrypted data with aggregated data, so as to complete the integrity of privacy data protection.

## 4. Analysis and Research of Intrusion Detection Technology Based on Particle Swarm Optimization Algorithm

**Figure 2:**
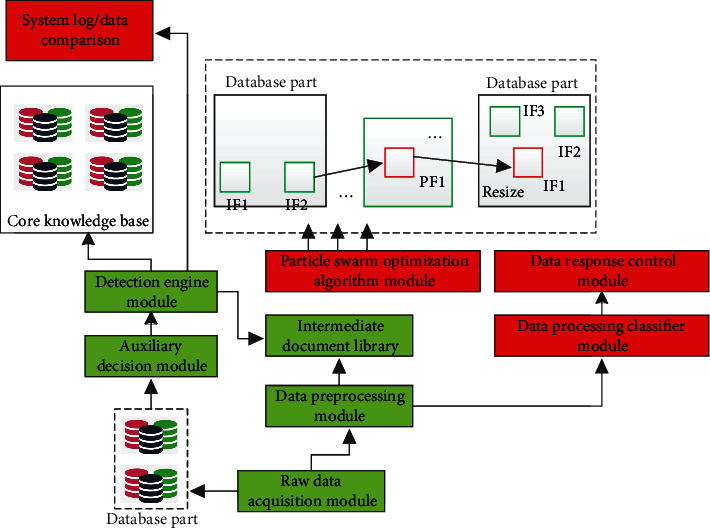
Technical framework of intrusion detection technology based on particle swarm optimization algorithm.

The intrusion detection technology mainly combines the artificial neural network algorithm to complete the intrusion detection technology of external attack objects. The corresponding main technical framework is shown in Figure 2. The main technical modules include network information data acquisition module, network data auxiliary decision-making module, preprocessing module, particle swarm optimization algorithm module, classifier generation module, detection engine module, and system response control module.

At the level of the corresponding network information data acquisition module, it obtains network data, refines the corresponding data, and processes and analyzes these preprocessed modules at the same time. To solve the analysis and processing of information and data packets transmitted on the network, so as to provide use and analysis for subsequent functional modules, at the level of the corresponding network data auxiliary decision-making module, it mainly takes the network data as the source of the data, then analyzes the access volume of each host in the network, and classifies and processes the corresponding host based on the weighted mean value and the corresponding traffic, so as to further guide the network information data to be shunted according to the destination address, and the processing of this module will provide corresponding auxiliary decision-making for data shunting; at the level of the corresponding preprocessing module, it is mainly to further process and analyze the original data, so the corresponding quality of the corresponding data preprocessing data will directly affect the final system decision-making level; at the level of the corresponding classifier generation module, it mainly detects and processes the preprocessed data and the nodes trained by artificial neural network; the function of the corresponding detection engine module is mainly to further judge, process, and analyze the preprocessed data, and judge whether the corresponding data are illegal data and foreign intrusion data. It is mainly to score the processed data with the information of the corresponding knowledge base, so as to further judge whether it is foreign attack information; the corresponding response control module is mainly for the response processing and analysis of the system. It mainly realizes three functions: real-time online monitoring of abnormal data of intrusion, real-time updating of data in knowledge base, and information communication and interaction with corresponding system administrators.

**Figure 3:**
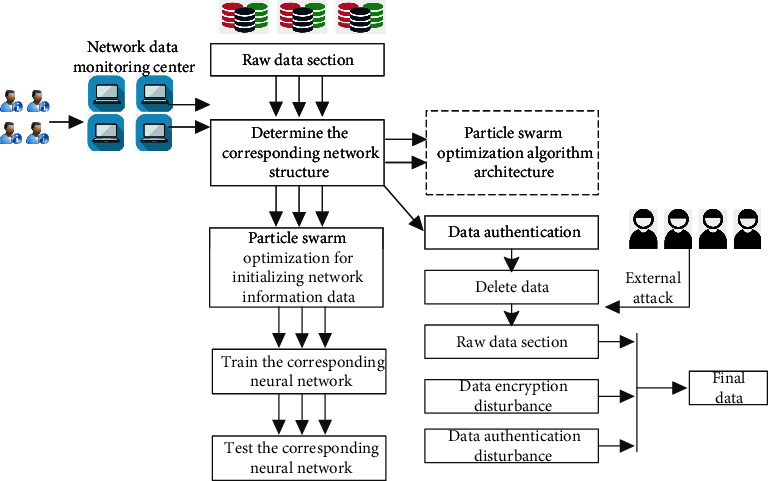
Core algorithm particle swarm optimization algorithm design idea frame diagram.

In the corresponding preprocessing and auxiliary decision-making levels, particle swarm optimization algorithm will be used to process the corresponding network information data. Therefore, the design idea of the corresponding core algorithm particle swarm optimization algorithm is shown in Figure 3. The following can be seen:

  Step 1: determine the corresponding network structure and the total number of all connection weights in the corresponding network. The corresponding number is the dimension of the solution vector represented by the particle swarm, and the dimension value of the corresponding solution vector corresponds to the connection value.
  Step 2: initialize the particle swarm optimization of network information data. The size of the corresponding particle swarm is determined, and the corresponding times of evolution, the corresponding particle velocity and the range of solution space, and the initial velocity and corresponding position of each corresponding particle are also recorded.
  Step 3: train the corresponding neural network. The solution vector represented by each particle in the corresponding particle swarm is mapped to the weight in the corresponding network, so as to construct the corresponding neural network. Here, in order to optimize the generalization ability of neural network, the collected data samples are divided into two parts for processing: one part is processed as training samples, and the corresponding part is processed as test samples. The input values of corresponding training samples are input into neural network, and then the actual output values are obtained through calculation.
  Step 4: test the corresponding network. The corresponding neural network is generated based on the corresponding weight, and the neural network is used to prepare for the generation of the following classifier.

## 5. User Privacy Protection Scheme Based on Polynomial Regression and Same State Encryption

**Figure 4:**
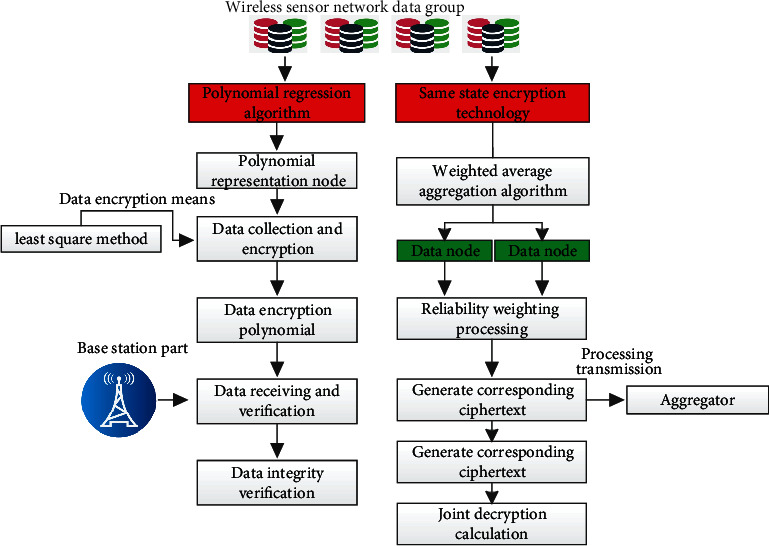
Flowchart of user privacy protection scheme based on polynomial regression and same state encryption.

In the corresponding wireless sensor network system, privacy protection is mainly based on polynomial regression algorithm and the same state encryption algorithm. The two algorithms cooperate, which is similar to double guarantee, so as to realize the privacy protection of wireless sensor networks. The corresponding algorithm flowchart is shown in Figure 4.

In the corresponding polynomial regression algorithm protection, firstly, the nodes in the wireless sensor network system are represented by polynomials. The corresponding representation formula is shown in formula (1), where the corresponding *a* and *b* represent the parameters of the system, the corresponding *n* represents the sensing data set of the system, and the corresponding *m* represents the order of the polynomial function.

(1) $$\begin{array}{c} \left[a,b\right]=a_{11}*\left[a,b\right]1+...+a_{mn}*\left[a,b\right]m. \end{array}$$

Based on the above formula, the data in its wireless sensor network are collected and encrypted. The main encryption method depends on the least square method. In practical application, multiple random numbers are used to encrypt the polynomial. The corresponding encryption polynomial expression formula is shown in formula (2). The corresponding mathematical symbol *f ()* is the encryption function expression, and the corresponding a and B represent the parameters of the system, which is consistent with the mathematical symbol shown in formula (1).

(2) $$\begin{array}{c} f_{1}\left(a,b\right)=\left(a_{11}+r_{1}^{j}\right)*{\left(a,b\right)}^{1}+...+\left(a_{ij}+r_{1}^{j}\right)*{\left(a,b\right)}^{j},\\ f_{x}\left(a,b\right)=\left(a_{11}+r_{1}^{j}\right)*{\left(a,b\right)}^{1}+...+\left(a_{ij}+r_{1}^{j}\right)*{\left(a,b\right)}^{j}. \end{array}$$

Based on the above polynomial encryption processing, the data transmission and aggregation are processed. The polynomial encrypted by random number itself has certain privacy. In the system proposed in this paper, the corresponding encrypted data are mainly generated by the corresponding nodes of the sensor, and external cannot obtain the key in essence. The sensor node continuously sends the corresponding encrypted coefficients and nodes to the aggregator terminal, and then the aggregator terminal will conduct corresponding aggregation, complete the aggregation within a specific time, and ensure that the corresponding sensor node transmits the data. The corresponding polynomial formula is shown in

(3) $$\begin{array}{c} M^{d}\left(a,b\right)=\text{Conf}\left(1,1\right)+\text{Conf}\left(...\right)+\text{Conf}\left(i,j\right). \end{array}$$

After completing the data transmission and aggregation, the corresponding base station completes the final data reception and verification. The base station will obtain the decrypted aggregated data and verify the integrity of the corresponding data, so as to verify the effectiveness of aggregation and finally complete the protection of relevant privacy.

After the polynomial regression processing is completed, the corresponding data are encrypted in the same state. The same state encryption processing scheme adopted in this paper is the weighted average aggregation algorithm. The algorithm finally realizes the corresponding privacy protection by weighting the reliability of the corresponding data nodes. The main process is as follows:

  Step 1: generate the corresponding ciphertext based on relevant data, and transfer the corresponding ciphertext to the aggregator for processing.
  Step 2: generate the ciphertext of the weight vector based on the terminal of the trusted institution. The main reason is that the weight vector will directly reflect the reliability of each data node in the wireless sensor network system. Therefore, the corresponding weight vector needs to be encrypted here.
  Step 3: encrypt the corresponding aggregate value and calculate its sum. In the actual calculation, bilinear mapping is mainly used to multiply the corresponding encrypted data.
  Step 4: perform joint decryption calculation based on trusted institutions and corresponding aggregators.
  Step 5: release the corresponding results to meet the differential privacy. The main responsibility of the corresponding trusted organization here is to release the final calculation results to the base station under the protection of differential privacy and finally complete the corresponding privacy protection.

## 6. Experimental Verification and Data Analysis

**Figure 5:**
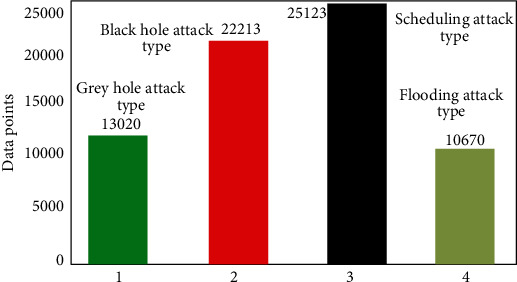
Distribution of attack types in wsn-ds experimental data set.

In order to verify the superiority of this algorithm, it is compared with the traditional algorithm. The main experimental data set is wsn-ds network intrusion data set, which contains a large number of network attacks, such as flooding attack, gray hole attack, scheduling attack, and black hole attack. Based on this, this paper makes distribution statistics on the attacks in the corresponding data set. The corresponding statistical data figure is shown in Figure 5 and Table 1. From Figure 5, it can be seen that there are more gray hole attacks and scheduling attacks in this data set and fewer ordered flooding attacks. In the actual experimental verification, this paper ensures the variable control and environment control between this algorithm and the traditional algorithm. Based on this, the corresponding indicators in the experimental part are described as follows: (1) accuracy: mainly used to test the type performance of known data and the performance of unknown data types; (2) database-based update performance: mainly used to test the system's internal known database performance; and (3) test performance under the influence of shunt: mainly used to detect the test performance under the influence of shunt.

Based on the above experimental data, this paper will start with four indicators in intrusion detection and privacy protection. The corresponding four levels are the ability test based on known intrusion data types, the test based on unknown data types, the test based on the update ability of the internal knowledge base of the system, and the test based on the influence of system diversion.

**Figure 6:**
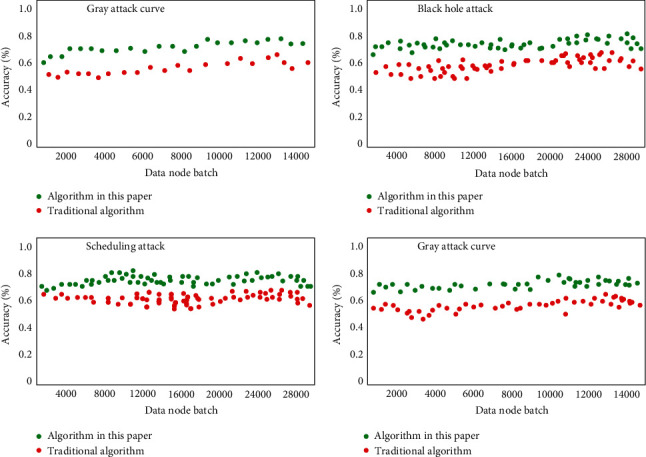
Capability test accuracy curve based on known data types.

At the level of corresponding capability test based on known data types, the intrusion test is mainly based on the classified data in the above wsn-ds data set. The corresponding test results are shown in Figure 6. It can be seen that it has advantages in the accuracy of corresponding intrusion recognition in various data types of attacks compared with the traditional algorithm. In some types of intrusion such as scheduling attack, the corresponding recognition accuracy is 11.2% higher, and the recognition accuracy corresponding to the worst gray hole attack data type is also about 2% higher.

**Figure 7:**
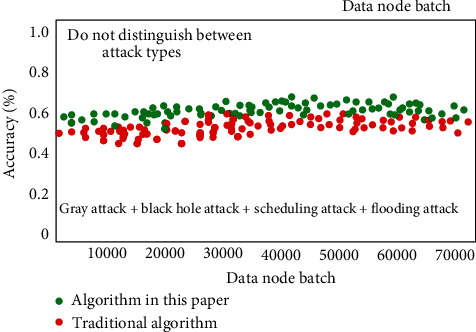
Test accuracy result chart based on unknown data type.

At the corresponding test level based on unknown data type, the corresponding data have no special label. In the actual test, this paper directly marks all the attack contents as intrusion data without specific classification. Based on this, the corresponding detection results are shown in Figure 7. It can be seen that the algorithm proposed in this paper can leave more data about the type of attack than the traditional algorithm, and the corresponding overall recognition rate is about 5% higher than the traditional algorithm.

**Figure 8:**
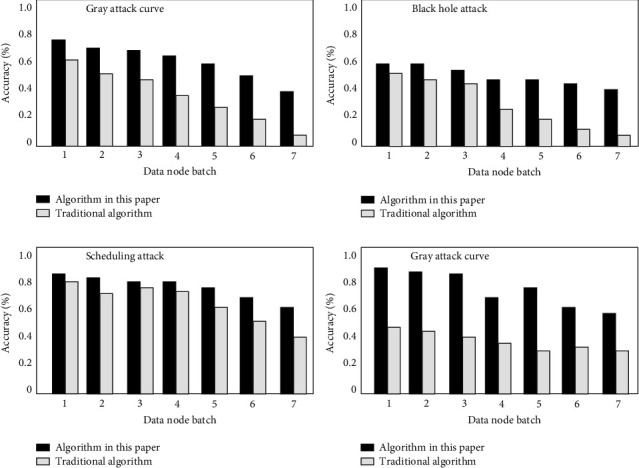
Result chart of updating capability test accuracy based on internal knowledge base of the system.

At the test level based on the update ability of the internal knowledge base of the system, the purpose of the experiment is mainly to verify whether the new data mode is added to the corresponding database in time, and whether the corresponding new detection engine module can effectively identify the corresponding intrusion data. Based on this, the corresponding test results are shown in Figure 8. It can be seen that the algorithm designed in this paper has better adaptability, which can quickly adapt to the update of the knowledge base. At the same time, the correct identification of the corresponding database intrusion will also be recorded in the knowledge base, so as to further increase the detection efficiency of the system.

**Figure 9:**
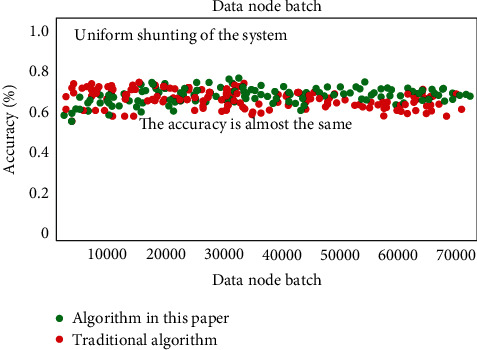
Test-level accuracy results based on the influence of system diversion (this experiment only considers the test accuracy results under the condition of uniform shunting, and there is no corresponding data support for nonuniform shunting).

At the corresponding test level based on the impact of system diversion, it is mainly to verify the adaptability of the system designed in this paper in the face of unbalanced traffic. The corresponding experimental results are shown in Figure 9. It can be seen that there are few errors in the misjudgment of normal data, but it has little gap with the traditional algorithm, and its recognition ability of corresponding sudden data is still insufficient. Therefore, it needs to be further strengthened in the follow-up research.

From the above theoretical analysis and the corresponding experimental data results, it can be concluded that the intrusion detection system proposed in this paper has obvious advantages compared with the traditional system, and has important value and significance for protecting the privacy of wireless sensor network system and detecting intrusion data in time. The above corresponding experimental results further verify the advantages of the relevant theories in this paper and also provide important support for the subsequent optimization and development of the theory.

## 7. Conclusion

This paper mainly analyzes the research status and existing problems of privacy protection and intrusion detection technology in wireless sensor network system. Based on this, this paper has done a lot of theoretical analysis and experimental work. Based on the traditional privacy protection and intrusion detection system, this paper makes an in-depth study on the wireless sensor network system based on neural network. Firstly, this paper applies the particle swarm optimization algorithm to the intrusion detection system and constructs the wireless sensor network intrusion detection system based on particle swarm optimization algorithm. The system includes important modules such as data extraction, data analysis, data feedback, and auxiliary decision-making; at the corresponding level of wireless sensor network privacy protection, based on the original data aggregation privacy protection scheme, this paper proposes a privacy protection scheme based on polynomial regression and a user privacy protection scheme based on the same state encryption, which further improves the security of privacy protection and facilitates the management of information. In order to realize the integrity of user privacy information protection, this paper realizes the decryption of data based on the correlation between binary metadata and compares the corresponding decrypted data with aggregated data, so as to complete the integrity of privacy data protection. The results show that the privacy protection and intrusion detection system designed in this paper have high practical value. In the subsequent research, this paper will focus on analyzing the recognition accuracy of the corresponding intrusion detection system in the case of unbalanced intrusion data and reasonably distinguish the normal system data. At the same time, this paper will also focus on solving the problem of system energy consumption and improving the efficiency of the algorithm in the later research.

## Figures and Tables

**Table 1:** Classification of attack types in the experimental data set.

Attack type	Gray hole attack type	Black hole attack type	Scheduling attack type	Flooding attack type
Quantity statistics	13020	22213	25213	10670
Experimental data set type	wsn-ds experimental data set			

## Data Availability

The data used to support the findings of this study are available from the corresponding author upon request.
